# Transorbital Approach for Endovascular Occlusion of Carotid-Cavernous Fistulas: Technical Note and Review of the Literature

**DOI:** 10.7759/cureus.976

**Published:** 2017-01-12

**Authors:** Ching-Jen Chen, Panagiotis Mastorakos, James P Caruso, Dale Ding, Paul J Schmitt, Thomas J Buell, Daniel M Raper, Avery Evans, Steven A Newman, Mary E Jensen

**Affiliations:** 1 Department of Neurological Surgery, University Of Virginia; 2 Department of Neurological Sugery, University Of Virginia; 3 Department of Interventional Neuroradiology, University Of Virginia; 4 Department of Ophthalmology, University Of Virginia

**Keywords:** transorbital, carotid cavernous fistula, fistula, endovascular, dural arteriovenous fistula, ccf

## Abstract

Carotid-cavernous fistulas (CCFs) pose an anatomically and physiologically challenging problem for clinicians. The most common method of treatment for these lesions is transvenous endovascular embolization via the inferior petrosal sinus or the facial vein. When transvenous access is not possible, an alternate approach must be devised. We describe a case example with bilateral Barrow Type B CCFs, which were inaccessible using the traditional transvenous approach. Hence, a direct transorbital approach, performed under fluoroscopic guidance, was employed to successfully obliterate the CCF. At five months follow-up, the patient was recovering without complications. This case delineates the technical aspects of transorbital CCF embolization and demonstrates that this approach is a viable alternative to conventional transvenous methods for appropriately selected CCF cases. We supplement our case example and technical note with a literature review of this approach.

## Introduction

A carotid-cavernous fistula (CCF) is an abnormal arteriovenous connection between the cavernous sinus (CS) and the cavernous segment of the internal carotid artery (ICA). The most common treatment modality for CCFs is endovascular embolization via transvenous catheterization. Numerous routes exist for obtaining transvenous access, including the inferior petrosal sinus and facial vein. However, rare cases arise in which anatomic constraints preclude transvenous access to the CS. In these patients, a direct transorbital approach may be employed to access and obliterate the fistula. The following technical note describes our procedural experience with transorbital embolization of a CCF.

## Technical report

**Figure 1:**
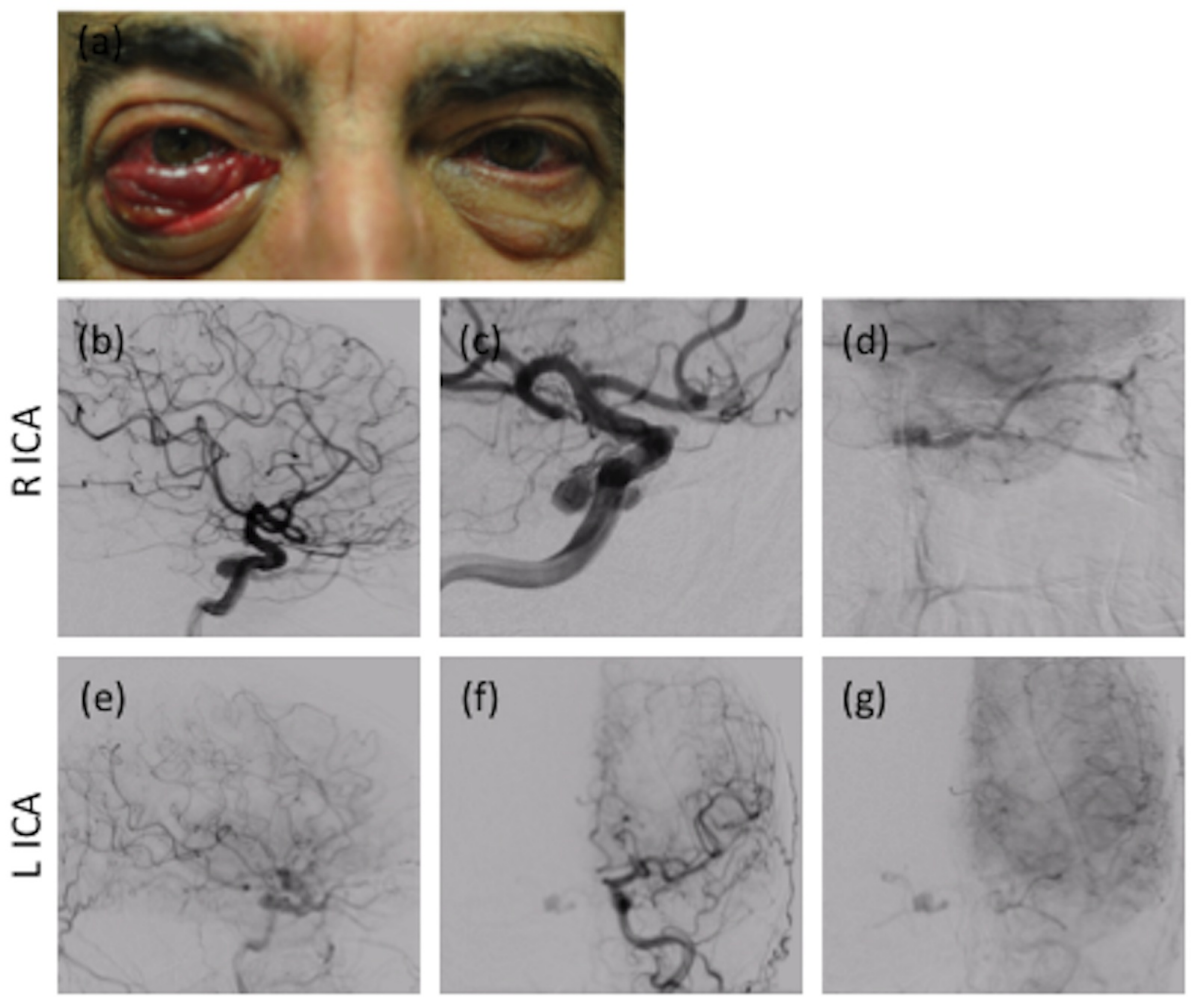
Initial Presentation and Diagnostic DSA (a) External examination demonstrating 2-3+ conjunctival injection and 2+ prominence of episcleral vessels of the right eye with 8 mm of exposed conjunctiva. (b-d) DSA following R ICA injection (b) lateral and (c) oblique views of intracranial circulation in arterial phase; (d) lateral view of intracranial circulation in venous phase. (e-g) DSA following L ICA injection (e) lateral view of late arterial phase; (f, g) AP view of (f) arterial and (g) capillary phase. Demonstration of right-sided, indirect, Type B CCF supplied by branches of the meningohypophyseal trunk, inferolateral trunk (b, c), and collaterals from the contralateral meningohypophyseal trunk (f, g), with venous outflow into the SOV and IOV and eventually into the FV (d). Demonstration of left-sided, indirect, Type B CCF supplied by smaller caliber branches of the meningohypophyseal trunk (e-g). DSA: digital subtraction angiography; L ICA: left internal carotid artery; R ICA: right internal carotid artery; AP: anteroposterior; CCF: carotid-cavernous fistula; SOV: superiot ophthalmic veins; IOV: inferior ophthalmic veins; FV: facial vein

A 68-year-old male presented to the ophthalmology clinic as a referral for thyroid orbitopathy with diplopia. On examination, the patient’s visual acuity was 20/20 in the right eye, with 2+ conjunctival injection and trace chemosis. In the left eye, his visual acuity was 20/30 with 2-3+ injection, 3+ chemosis, and prolapsed conjunctiva. A fundoscopic exam did not demonstrate disc edema, optic atrophy, or abnormalities in venous pulsation. Thyroid studies were normal, and magnetic resonance imaging (MRI) and angiography (MRA) demonstrated no evidence of a cavernous carotid fistula. The patient’s chemosis and injection improved with initial treatment using eye drops. The patient returned later to the ophthalmology clinic with 2-3+ conjunctival injection and 2+ prominence of episcleral vessels of the right eye with 8 mm of exposed conjunctiva (Figure 1a). A temporary tarsorrhaphy was performed to prevent keratinization of the exposed conjunctiva. The patient subsequently underwent a diagnostic cerebral angiogram, which showed bilateral CCFs. Informed patient consent was obtained for his treatment. No identifying patient information is contained within this report.

### Technical details

**Figure 2:**
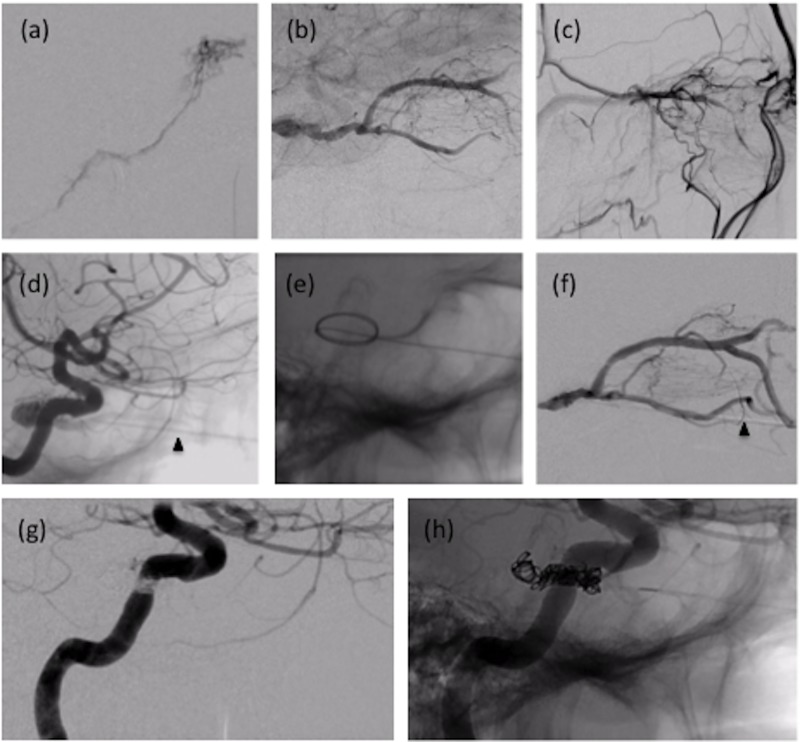
Therapeutic DSA (a) Lateral view of R inferior petrosal sinus demonstrating relatively normal appearance of the median portion of the CS, isolated from the CCF. (b, c) Lateral view demonstrates venous outflow anatomy of CCF and anatomy of peri-orbital cortical veins via contrast administration in R ICA (venous phase) (b) and facial vein (c). (d) Fluoroscopic demonstration of direct needle placement in IOV through DSA via R ICA injection. Arrowhead demonstrated needle. (e) Micro-guidewire advancement and coiling into the CS. (f) Assessment of venous outflow through direct contrast administration in CS. Arrowhead demonstrates catheter. (g, h) Successful coiling of R CS with no residual shunting of outflow observed from the R ICA to the CCF. CS: cavernous sinus; CCF: carotid-cavernous fistula; DSA: digital subtraction angiography; IOV: inferior ophthalmic veins; R ICA: right internal carotid artery

ICA injections demonstrated bilateral Barrow Type B CCFs (Figures 1b, 1g). The right fistula was supplied by branches of the meningohypophyseal trunk (MHT), the inferolateral trunk (ILT) (Figures 1b-1c), and collateral supply from the contralateral MHT (Figures 1f-1g). Venous efflux was through the superior (SOV) and inferior ophthalmic veins (IOV) into the facial vein (Figure 1d). The left fistula was supplied by branches of the MHT, with venous efflux also through the SOV and IOV into the facial vein (Figures 1e, 1g). The left-sided fistula was relatively small in caliber, and we felt it would thrombose without intervention; therefore, the decision was made to only embolize the right-sided CCF. An initial attempt was made to access the CCF via the right inferior petrosal sinus (IPS). However, digital subtraction angiography (DSA) demonstrated that the medial portion of the CS connected to the IPS was isolated from the CCF (Figure 2a). Under roadmap guidance, the diagnostic catheter was advanced through the external jugular vein into the right facial vein over a guidewire. Venous outflow anatomy of the CCF and anatomy of the peri-orbital cortical veins were assessed via contrast administration through the right ICA (venous phase) (Figure 2b) and facial vein (Figure 2c). Multiple attempts were made to access the CCF venous outflow tract using a Marksman™ micro catheter (ev3 Neurovascular, Plymouth, MN), Echelon™ 14 micro catheter (ev3 Neurovascular), Transend-EX® .014 micro-guidewire (Stryker, Kalamazoo, MI), and Synchro2® micro-guidewire (Stryker) without success. Without a transvenous route into the right CCF, the decision was made to directly access the CS through a transorbital approach.

The venipuncture was performed using a 21G needle. The needle was advanced along the floor of the right orbit, and the IOV was accessed at the inferior orbital fissure (Figure 2d). A micro-guidewire was advanced through the needle and coiled within the CS (Figure 2e). The needle was then exchanged over the micro-guidewire for a 4Fr dilator. The wire was removed and contrast was injected under fluoroscopy to verify positioning (Figure 2f). The dilator was then sutured in place, and a rotating hemostatic valve (RHV) and stopcock connected to continuous heparinized saline flush were connected to the dilator. A 5Fr diagnostic catheter was then advanced into the RHV and married to the hub of the dilator, allowing the operator to perform the coil embolization away from the radiation field. The CS was initially framed with an Axium™ 3 mm x 8 cm coil (ev3 Neurovascular). Next, the dilator was retracted slightly, and the CCF was further embolized using two Axium 2 mm x 6 cm coils, one Axium™ 3 mm x 8 cm coil, one Axium™ 2 mm x 8 cm coil, and one HydroSoft® Advanced 2 mm x 6 cm Helical Coil (MicroVention, Tustin, CA). These coils were submerged in thrombin prior to deployment to promote thrombosis. Post-embolization angiography demonstrated no significant residual shunting from the right ICA through the CCF (Figures 2g-2h).

### Follow-up

**Figure 3:**
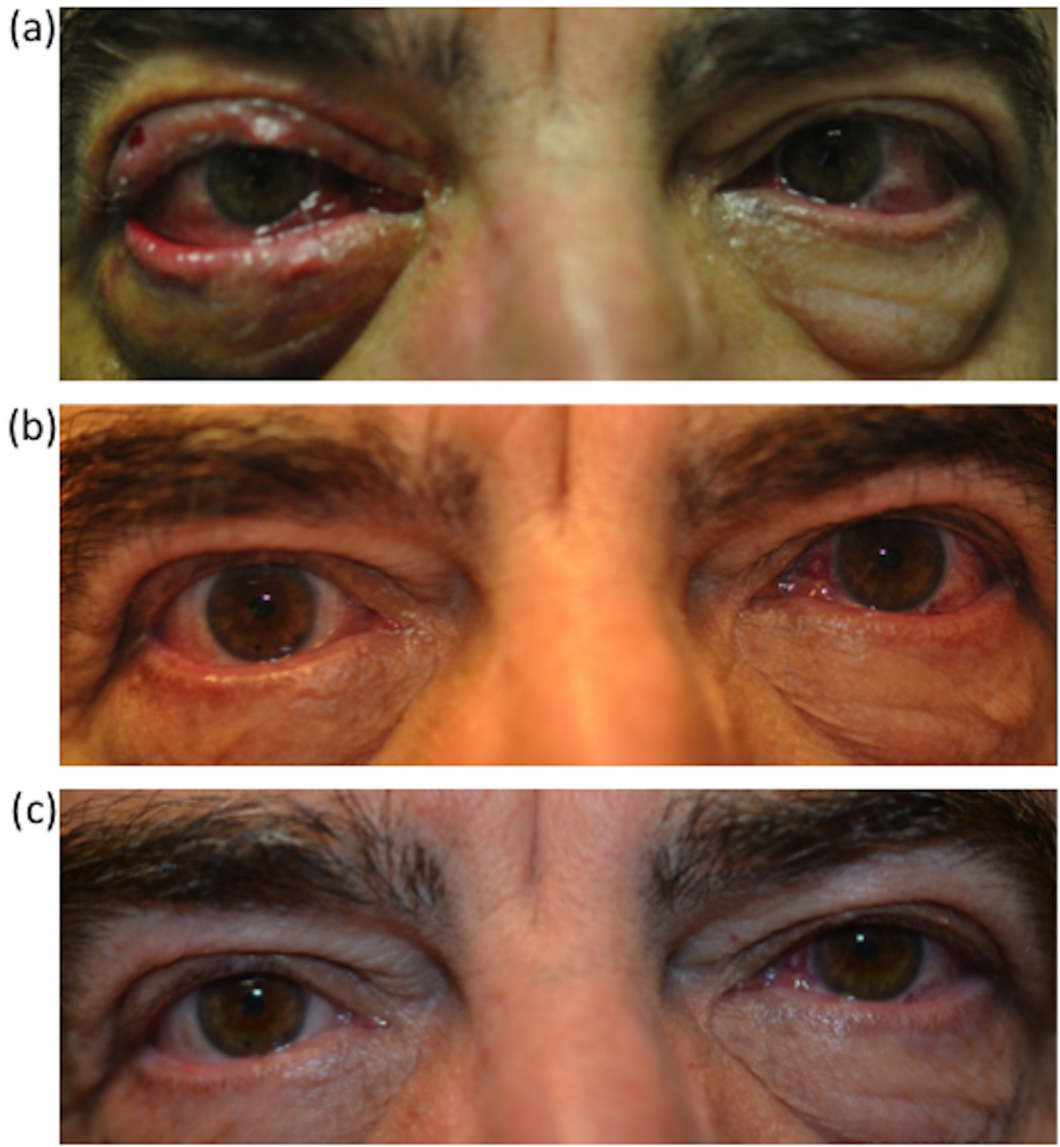
Ophthalmology Follow-up Images (a) Three day ophthalmology follow-up reveals improved chemosis and conjunctival injection, along with a steadily improving partial CN III and CN IV palsy. Follow-up at six weeks (b) demonstrated complete resolution of the arterialized vessels in the right eye and improvement in the CN III and IV palsies. Follow-up at five months (c) demonstrated only mild CN III and IV palsies. CN: cranial nerve

Ophthalmologic examination three days after embolization demonstrated a marked decrease in chemosis after reversal of the tarsorrhaphy (Figure 3a). The patient had an interval development of right cranial nerve (CN) III and IV palsies. Follow-up at six weeks demonstrated complete resolution of the arterialized vessels in the right eye and improvement in the CN III and IV palsies (Figure 3b). Subsequent follow-up at five months demonstrated only mild CN III and IV palsies without diplopia or evidence of progressive optic neuropathy (Figure 3c). 

## Discussion

CCFs consist of a group of vascular malformations characterized by an aberrant shunt between one or more sources of arterial inflow and the CS; they are subdivided into direct and indirect fistulas [1]. Direct CCFs, which involve a primary connection between the ICA and CS, typically result from trauma or aneurysm rupture and often present with proptosis, chemosis, orbital bruit, and visual disturbances. Traumatic CCFs are most common in young males. They occur in 0.2% of head injuries and in 4% of basilar skull fractures [2]. Following trauma, a CCF may develop due to a tear in the carotid artery from an external force or as a result of vessel rupture from increased intraluminal pressure combined with compression of downstream vessels [2]. Spontaneous CCFs occur most commonly in older female patients. They can result from ruptured ICA aneurysms as well as genetic conditions, such as fibromuscular dysplasia and Ehlers-Danlos syndrome [2]. Indirect CCFs involve fistulous connections between branches of the ICA or the external carotid artery (ECA). Their origin is less well understood and is thought to be associated with venous outflow obstruction during development. While indirect CCFs have a more indolent course of progression and often present with conjunctival injection, severe ophthalmologic complications may occur in cases with substantial venous outflow obstruction [3]. The Barrow classification scheme is the most widely used system for the categorization of CCFs (Table 1).

**Table 1:** Barrow Classification ICA: internal carotid artery; CS: cavernous sinus; ECA: external carotid artery

Type	Description
A	Direct connection between ICA and CS
B	Dural shunt (indirect) between meningeal branches of ICA and CS
C	Dural shunt (indirect) between meningeal branches of ECA and CS
D	Dural shunt (indirect) between meningeal branches of the ICA, ECA, and CS

Endovascular techniques are the mainstay approach for the treatment of CFFs, and microsurgical treatment is currently utilized when the endovascular approach has failed [2]. Tu, et al. presented a series of 78 patients treated with direct surgical obliteration of CCF [4]. A variety of surgical methods were used, including clipping and sealing the CCF with fascia and acrylate glue. All but one patient in the study had undergone endovascular embolization prior to surgical treatment. The CCF obliteration rate was 100%, and the most common postoperative morbidity was transient ocular palsy. Day, et al. presented a series of nine patients who underwent surgery for CCF following the failure of embolization [5]. All patients experienced symptomatic relief, there were no deaths, and transient ocular complications resolved by six months follow-up. Stereotactic radiosurgery has also shown promise as both a primary and adjuvant treatment for CCFs. A systematic review by Chen, et al. found that radiosurgical treatment of CCFs resulted in a 73% obliteration rate with no post-SRS hemorrhage. The study also noted that Barrow Type A CCFs are less amenable to radiosurgical occlusion.  Endovascular treatment proves to be a timely and successful treatment modality for Barrow Type A CCFs, while select patients with Types B, C, and D CCFs may benefit from radiosurgery [6]. 

Despite successful reports of CCF treatment with surgery and radiosurgery, endovascular embolization remains the first-line therapy. Transarterial approaches are most commonly used for direct CCFs, while both transarterial and transvenous approaches are utilized for indirect CCFs. The transarterial approach for direct CCFs involves accessing the fistula via the ICA. For indirect CCFs, it involves distal access via feeding vessels, usually from the ECA, an approach that is fraught with difficulty [7]. Transvenous strategies are particularly useful for indirect CCFs with small ICA feeders. The heterogeneity of venous drainage amongst indirect CCFs requires an adaptive approach, depending on the venous outflow patterns. The inferior petrosal sinus is commonly used to access the venous system, especially in cases with predominantly posterior drainage. Meyers, et al. reported a 76% success rate when approaching the CS via the inferior petrosal sinus. Similarly, Klisch, et al. were able to completely or partially occlude 60% of CCFs using this approach. When the inferior petrosal sinus is opacified, thrombosed, or otherwise not angiographically visible, a transvenous approach via the facial vein is an alternative. Klisch, et al. reported a 50% treatment success rate when the facial vein was used to access the CS [8]. A case series of seven patients by Biondi, et al. analyzed the success of endovascular treatment following access to the CS via the facial vein [9]. In all seven patients, the CS was successfully accessed through the angular vein and the superior ophthalmic vein. Catheterization through the superior ophthalmic vein failed in one patient. Four patients demonstrated complete CCF occlusion, and two additional patients improved clinically.

Despite the success of the above strategies, complex angioarchitecture can preclude conventional transarterial or transvenous approaches. The transorbital approach is a viable alternative for endovascular treatment of CCFs. This technique allows access to the CS through the SOV [10-13], IOV [11], or ICA [14-15] or through a direct transorbital puncture of the CS [15-20]. The reported case demonstrates the use of the transorbital approach for coil embolization of a Barrow Type B CCF. A review of the literature identified 12 case reports of the transorbital approach for CCFs (Table 2). Observed complications included postoperative ptosis, proptosis, chemosis, and CN palsies [10, 12-13, 15-16]. Teng, et al. observed that other potential complications include subarachnoid hemorrhage, vision loss, and optic nerve injury [15]. Workman, et al. posit that subarachnoid hemorrhage can be avoided by entering the CS anteriorly, thereby avoiding a breach of the subarachnoid space [16]. Elhammady, et al. reported success using an Onyx® ethylene vinyl alcohol copolymer (EV3 Neurovascular, Irvine, CA) embolization. Onyx allowed the authors to target the posterior compartment of the CCF containing the point of fistulization. The use of coils would have likely led to compartmentalization and incomplete obliteration of the CCF [18]. Mehrzad, et al. also demonstrated success using Onyx embolization [10], and Dashti, et al. demonstrated success using a combination of Onyx and detachable coils [11].  

**Table 2:** Reports of CCF Treatment Via Transorbital Approach CCF: carotid-cavernous fistula; CN: cranial nerve; N/A: not available

Article	Patients (#)	Barrow Type	Result (#)	Complications (#)
Teng, et al. 1995 [15]	11	A (11)	Complete obliteration (11)	Transient postoperative ptosis (2)
Workman, et al. 2002 [16]	1	A (1)	Complete obliteration	Transient postoperative ptosis, proptosis, and chemosis
Satchi, et al. 2009 [17]	1	D (1)	Complete obliteration	None
Elhammady, et al. 2011 [18]	1	B(1)	Complete obliteration	None
Mehrzad, et al. 2011 [10]	1	C (1)	Complete obliteration	Complete CN III palsy (resolved at 3 months)
Dashti, et al. 2011 [11]	2	B (1), D (1)	Complete obliteration (2)	None
Luo, et al. 2013 [14]	1	D (1)	Complete obliteration	None
Pansara, et al. 2013 [12]	1	D (1)	Complete obliteration	Transient diplopia, proptosis, chemosis, and CN VI palsy
Coumou, et al. 2013 [19]	1	N/A (Indirect, low-flow CCF)	Complete obliteration	None
Puffer, et al. 2014 [20]	1	B (1)	Complete obliteration	None
Milburn, et al. 2014 [13]	1	D (1)	Complete obliteration	Transient CN VI palsy, proptosis, diplopia

## Conclusions

CCFs are most commonly managed with endovascular embolization. The clinical experience of the authors, along with a review of current literature, reveals that, for CCFs, which are inaccessible from a transvenous approach, direct transorbital embolization is a safe and effective alternative for occlusion of these lesions.
